# Motivation and satisfaction among community health workers in Morogoro Region, Tanzania: nuanced needs and varied ambitions

**DOI:** 10.1186/s12960-015-0035-1

**Published:** 2015-06-05

**Authors:** Rose N M Mpembeni, Aarushi Bhatnagar, Amnesty LeFevre, Dereck Chitama, David P Urassa, Charles Kilewo, Rebecca M Mdee, Helen Semu, Peter J Winch, Japhet Killewo, Abdullah H Baqui, Asha George

**Affiliations:** Muhimbili University of Health and Allied Sciences, School of Public Health and Social Sciences, P.O. Box 65015, Dar-es-Salaam, Tanzania; Department of International Health, Johns Hopkins Bloomberg School of Public Health, 615 North Wolfe Street, Baltimore, MD 21205 USA; Jhpiego, Bagamoyo Road, Victoria Area, P.O. Box 9170, Dar es Salaam, Tanzania; Tanzania Ministry of Health and Social Welfare, Health Promotion Section, 6 Samora Machel Avenue, 11478 Dar es Salaam, Tanzania

**Keywords:** Community health worker, Motivation, Satisfaction, Non-financial incentives, Financial incentives

## Abstract

**Background:**

In 2012, the Ministry of Health and Social Welfare (MOHSW), Tanzania, approved national guidelines and training materials for community health workers (CHWs) in integrated maternal, newborn and child health (Integrated MNCH), with CHWs trained and deployed across five districts of Morogoro Region soon after. To inform future scale up, this study assessed motivation and satisfaction among these CHWs.

**Methods:**

A survey of all CHWs trained by the Integrated MNCH Programme was conducted in the last quarter of 2013. Motivation and satisfaction were assessed using a five-point Likert scale with 29 and 27 items based on a literature review and discussions with CHW programme stakeholders. Exploratory factor analysis was conducted to identify motivation and satisfaction determinants.

**Results:**

Out of 238 eligible CHWs, 96 % were included in the study. Findings showed that respondents were motivated to become CHWs due to altruism (work on MNCH, desire to serve God, work hard) and intrinsic needs (help community, improve health, pride) than due to external stimuli (monetary incentives, skill utilization, community respect or hope for employment). CHWs were satisfied by relationships with health workers and communities, job aids and the capacity to provide services. CHWs were dissatisfied with the lack of transportation, communication devices and financial incentives for carrying out their tasks. Factors influencing motivation and satisfaction did not differ across CHW socio-demographic characteristics. Nonetheless, older and less educated CHWs were more likely to be motivated by altruism, intrinsic needs and skill utilization, community respect and hope for employment. Less educated CHWs were more satisfied with service and quality factors and more wealthy CHWs satisfied with job aids.

**Conclusion and recommendations:**

A combination of financial and non-financial incentives is required to support motivation and satisfaction among CHWs. Although CHWs joined mainly due to their altruistic nature, they became discontented with the lack of monetary compensation, transportation and communication support received. With the planned rollout of the national CHW cadre, improved understanding of CHWs as a heterogeneous group with nuanced needs and varied ambitions is vital for ensuring sustainability.

## Background

Tanzania, like many other low-income countries, is experiencing chronic shortages of facility-based healthcare providers. It has four health professionals, including physicians, nurses and midwives, for every 10 000 people [1], in contrast to the 25 health professionals per 10 000 people recommended by WHO for achieving adequate coverage of critical maternal, newborn and child health (MNCH) interventions [2]. This acute shortage of health workers in Tanzania is stalling improvements in MNCH outcomes and threatening the country’s potential for achieving the Millennium Development Goals (MDGs).

The extreme shortages in human resources for health and the need to rapidly increase access to services led to a reconsideration of cadres such as community health workers (CHWs) in health systems. A CHW can be defined as any health worker carrying out functions related to healthcare delivery, trained in some way to deliver an intervention and having no formal professional or paraprofessional certificate or tertiary education degree [3]. CHWs can be deployed to create demand for health services, support linkages to facilities and in some instances provide basic health services. In countries where effective CHW programmes are in existence, studies show that CHWs are successful in improving health service coverage, continuity of care and health outcomes [3–7].

Despite the positive effects of CHW programmes, sustainability of these programmes remains a concern. Attrition rates range from 3.2 % to 77 % across different settings [8]. High CHW-attrition rates lead to discontinuity in service provision, increased costs to recruit and train new CHWs and lost opportunities to build on work experience, resulting in diminished effects on health outcomes [8]. Key factors contributing to attrition are low health worker motivation and satisfaction [9].

Motivation can be defined as the “willingness to exert and maintain an effort towards organizational goals” and is regarded to develop in individuals as a result of the interaction between individual, organizational and cultural determinants [10]. Job satisfaction is highly interrelated but distinct from motivation and is referred to as “*a pleasurable or positive emotional state resulting from the appraisal of one’s job or job experience*” [11].

Several factors influence motivation and job satisfaction among healthcare providers. Provision of financial incentives (including the possibility of future paid employment) and non-financial incentives are important for CHWs [8, 12]. Non-financial incentives include community recognition and respect, acquisition of valued skills, personal growth and development, accomplishment, peer support and community factors like relations with communities and leaders [13]. Other supportive CHW programme factors include frequent supervision and continuous training [12].

Motivation and satisfaction are key not only for retention but also for performance. Better workforce performance is positively associated with higher job satisfaction, and low levels of job satisfaction adversely affect employee commitment and sequentially affect achievement of organizational objectives and performance [14].

### National context

In Tanzania, the use of CHWs dates back to the mid-1960s when medical auxiliaries and village medical helpers were trained to run health posts. This emphasis on promoting access to health services was reinforced in 1978 after the Alma Ata declaration, and in 1983, the Ministry of Health and Social Welfare (MOHSW) developed a guideline for training primary healthcare workers in every village. Thereafter, a number of NGO-led vertical programmes resulted in heterogeneous CHWs often working in an uncoordinated manner. The lifetime of these CHW programmes was quite short, with a median of 4 years, though programmes with dedicated financial support and/or a narrow disease focus had more longevity [15].

In recent efforts to accelerate progress towards achieving MDGs 4 and 5, the Government of Tanzania laid a greater emphasis on the role of CHWs as articulated in the Primary Health Care Services Development Programme (2007–2017) and the National Road Map Strategic Plan to Accelerate Reduction of Maternal, Newborn and Child Deaths (one plan) [14, 15]. In 2012, national guidelines and training materials were approved for Integrated MNCH CHWs [16], with a pilot in Morogoro Region initiated soon after.

CHWs in this Integrated MNCH Programme are selected by village leaders through village meetings in collaboration with government health facility personnel using the following criteria: (1) at least a form IV education level, (2) willingness to work and live at the community level, (3) willingness to work on a voluntary basis and (4) acceptability by communities. After 3 weeks of training, the selected CHWs undertake antenatal and postnatal home visits to women of child-bearing age for health and nutrition counselling, early detection of pregnancy, recognition of danger signs and support for seeking facility-based antenatal, delivery and postnatal care. They also follow up newborns and children under 5 years with health promotion and preventive healthcare.

This study builds on prior qualitative research examining the motivation of CHWs in Morogoro Region [17] before being selected and trained for the Integrated MNCH Programme. This study examines CHW motivation and satisfaction quantitatively across a larger number of CHWs and in light of a specific programme. The Government of Tanzania is in the process of developing a national cadre of CHWs who are expected to bridge the gap between communities and the health system. The results from this pilot programme aim to inform programme planners and their efforts to develop appropriate strategies to ensure sustainability of CHW programmes in Tanzania and beyond.

## Methods

### Measuring motivation and satisfaction

Motivation and satisfaction are latent constructs, that is, they cannot be observed or measured directly. This study used two separate quantitative scales with 29 and 27 “measurable” items representing factors of motivation and job satisfaction, respectively. The true score or value of a latent construct is presumed to cause an item or set of items to take on a certain value [18]. However, given that the true score of a latent variable is not known, the correlations among items are used to infer how each item is correlated with the latent variable. These correlations between scale items and latent constructs are measured using a statistical technique called factor analysis. This method also allows for item reduction, by reducing redundancy or duplications, from a set of correlated items to a set of derived factors [19].

The scales used for measuring factors of motivation and job satisfaction were developed based on an extensive literature review and discussions with planners, managers and other stakeholders of the Integrated CHW programme. Motivational determinants were assessed using a five-point Likert scale including various reasons for becoming a CHW such as, among others, desire to help the community, opportunity to use skills, recognition for their services, lack of employment avenues and availability of financial incentives. Factors of CHW satisfaction were measured using a five-point Likert scale with items pertaining to training and supervision, availability of equipment and supplies, interpersonal relationships at work and remuneration.

### Study setting

The study was conducted in late September to November 2013 in five districts of Morogoro Region, Tanzania, where the Integrated MNCH Programme was implemented. Morogoro is the second largest and sixth most populous of Tanzania’s 25 regions. It lies 200 km west of Dar es Salaam with a population of 2.22 million people across 70 000 km^2^ [20]. With 73 % of its population living in rural areas, the region experiences averages similar to national averages for education and poverty [21].

#### Study population

All MNCH CHWs that received training by MOHSW/Jhpiego for the Integrated MNCH Programme at least 3 months prior to data collection (by July 2013) in the five administrative districts of Morogoro Region (Morogoro DC, Mvomero, Kilosa, Gairo and Ulanga) were eligible for inclusion in the study.

#### Data collection methods

Data collection was done by a team of five research assistants (RAs), fluent in Swahili, with graduate-level training in education, public health or social sciences. They were trained for 1 week by faculty from Muhimbili University of Health and Allied Sciences (MUHAS) and Johns Hopkins School of Public Health (JHSPH). Training included modules on study objectives, data collection methods and research ethics and was complimented by 2 days of pilot testing, immediately followed by data collection.

Jhpiego provided RAs with telephone numbers of the facility-based CHW supervisors who were contacted in advance to be informed of the survey. They were requested to inform CHWs under their supervision about the study and the date of the interviews. Data were collected over a maximum of 3 days in each village depending on the number and availability of CHWs in the village. Among consenting participants, RAs conducted interviews in participants’ homes in order to maintain privacy, as well as to directly observe indicators of socio-economic status.

Consenting CHWs were interviewed in Swahili for approximately 60 min to 90 min and asked to provide details about their socio-demographic characteristics and answer questions with unprompted response categories covering training and knowledge, as well as questions in a Likert scale for satisfaction and motivation. Efforts to ensure data quality were led by two field-based supervisors that provided support and were overseeing field implementation, including daily instrument review and conduct of daily debriefings with the data collectors.

### Data management and analysis

Completed surveys were transported to Dar es Salaam for data entry and cleaning every 2 weeks during data collection. Data were double entered and cleaned using Epi Info software at MUHAS in Dar es Salaam, Tanzania. Statistical analyses were performed using Stata 13.0. Descriptive data analysis was done to generate frequencies and trends across predictor variables. Given that the objective of this study was to identify factors of job satisfaction and motivation, and not confirm any existing theoretical models pertaining to the two constructs, exploratory factor analysis was conducted. In order to do so, principal component factor extraction and Promax rotation were used to identify factors that determine CHW job satisfaction and motivation by consolidating scale items into sets of derived factors. To determine the appropriate number of factors, four criteria were used: Kaiser-Guttman criterion of eigenvalue >1 [22], scree test [23], percentage of variance explained by the domain >5 % [24] and factor loading equal or greater than 0.4 [24]. Mean scale scores were generated for each factor, scaled out of 100 and standardized. Analysis of variance was used for identifying differences in reported factors of job satisfaction and motivation between sex, age groups, education and wealth levels among participating CHWs.

### Ethical considerations

The study protocol received ethical clearance at MUHAS and JHSPH Institutional Review Boards. Permission to conduct the study was obtained from regional, district and local authorities before the start of data collection. Respondents were informed of the study objectives along with risks and benefits of their participation. Interviews were conducted only after receiving written informed consent from CHWs. Confidentiality of information collected was ensured by not writing names of respondents on data collection forms and storing data on password-protected computers.

## Results

### MNCH CHW characteristics

A total of 228 of 238 (96 %) CHWs trained by the Integrated MNCH Programme prior to the study were interviewed with a median age of 32 (Table 1). Among respondents, 55 % were males, 51 % had completed secondary education and 60 % were married. The median household income among respondents was 50 000 TZS (US$ 29) per month, and the great majority (94 %) relied on farming as the main source of livelihood.

**Table 1:** Characteristics of 228 CHWs working for the Integrated MNCH Programme in Morogoro Region, Tanzania, in 2013

Characteristic	Results
Sex	
Female	45 %
Age (years)	
Median (range)	32 (19–61)
<25	30 %
25–35	33 %
>35	37 %
Marital status	
Married	60 %
Not married	32 %
Once married	8 %
Level of education	
Primary started but not completed	2 %
Primary education completed	47 %
Secondary education completed	51 %
Languages spoken	
Swahili	100 %
Tribal	84 %
English	15 %
Source of livelihood (not mutually exclusive)	
Agriculture (crops or livestock)	94 %
Non-agriculture	14 %
Wealth index mean (SD)^a^	
Lowest	−1.4 (0.47)
Middle	−0.12 (0.41)
Highest	1.82 (1.15)

^a^Principal component analysis was used to place CHWs in to three wealth quantiles based on ownership of selected assets, materials used for housing construction and access to water and sanitation facilities

### Motivation

Exploratory factor analysis of motivational determinants scale (Table 2) yielded four factors labelled as i) extrinsic push; ii) skill utilization, respect and hope; iii) altruism; and iv) intrinsic needs, which together explained about 62 % of total variance.

**Table 2:** Exploratory factor analysis of motivational determinants for 228 CHWs working for the Integrated MNCH Programme in Morogoro Region, Tanzania, in 2013

Reasons for becoming a CHW	% agreed	Factor 1: extrinsic push	Factor 2: skills utilization, community respect, hope for employment	Factor 3: altruism	Factor 4: intrinsic needs
I can continue working regardless of my age	72	0.663			
Job gives greater autonomy	66	0.828			
I can complete tasks without being told what to do	65	0.519			
My family has asked me to volunteer	40	0.657			
I am excused from shared community responsibility commitments	35	0.553			
Financial incentives received	4	0.553			
This work gives me respect in the community	78		0.405		
This job gives me the opportunity to use my skills	66		0.536		
For hoping to get more formal job from the government	45		0.511		
There are no other employment opportunities	37		0.778		
I am proud to work with the Integrated MNCH CHW Programme	97			0.560	
I want to make my community better	97			0.638	
This work gives me the opportunity to improve the health of others	91			0.757	
I want to help my fellow neighbours	82			0.565	
This work is focused on maternal and child health	90				0.475
By serving my community, I am serving God	86				0.593
I am a hard worker	70				0.790
% variance explained		21.95	16.08	13.01	10.66
Cronbach’s alpha		0.76	0.69	0.58	0.50
Mean out of 4		1.89	2.11	3.31	3.12
SD		0.06	0.06	0.04	0.05

The first factor was a varied combination of extrinsic stimuli including incentives received, autonomy at work, influence of family and job security. With the exception of a very few, most respondents (96 %) did not consider becoming a CHW due to the financial incentives offered. Among extrinsic stimuli, CHWs valued autonomy and job security in terms of lack of an age criteria as key reasons for becoming a CHW. On the whole, a mean score of 1.89 out of 4 for this factor suggests that most CHWs were less likely to be motivated by external stimuli.

The second factor included items pertaining to opportunities to use skills, respect received from the community attributed to the job and the potential of employment prospects. While a large proportion of CHWs were motivated by receiving community respect (78 %) and the opportunities to use skills (66 %), fewer CHWs chose to become health workers in the hope of formal government positions (45 %) or due to lack of other employment options (37 %). A mean score of 2.11 out of 4 for this factor suggests that it was more important than external stimuli but still only moderate.

In contrast to the first two factors, the other two factors generated from the motivational determinants scale had relatively high mean scale scores of 3.31 (altruism) and 3.12 (intrinsic needs) out of 4. Altruism corresponded with items on helping the community and improving its health, as well as pride in performing these services. Almost all respondents (82–97 %) reported to be motivated by altruism. The fourth factor included items pertaining to other intrinsic needs such as opportunity to serve God, working on maternal and child health and ability to perform tasks diligently, which was also reported as important by many CHWs (70–90 %).

Mean scale scores for motivation determinants were not significantly different between male and female CHWs. Similarly, no statistical difference was found between socio-demographic characteristics for the “extrinsic push” factor. On the other hand, mean scores for the remaining three factors (altruism; intrinsic needs; skill utilization, respect and hope) were found to be significantly higher for less educated and older CHWs. Mean score for the “skill utilization, respect and hope” factor was found to be greater for the relatively less wealthy respondents.

### Satisfaction

Exploratory factor analysis of satisfaction according to the job attributes scale (Table 3) resulted in five factors being identified in positive and negative ways. These included the following: i) availability of job aides and registers, ii) capacity to provide services and support received, iii) training and relations at work, iv) availability of transportation and v) provision and quality of services, which altogether accounted for 63 % of total variance.

**Table 3:** Exploratory factor analysis of job satisfaction for 228 CHWs working for the Integrated MNCH Programme in Morogoro Region, Tanzania, in 2013

	% Satisfied	Factor 1: job aides	Factor 2: capacity and support	Factor 3: job relations	Factor 4: transport	Factor 5: services and quality
Monthly report register	92	0.904				
Mother, newborn and child health register	91	0.876				
Referral register	91	0.902				
Availability of the job aides	90	0.400				
Own capacity to meet the health services needs of the community	81		0.747			
Own knowledge competency to perform work	71		0.488			
Support provided by facility supervisor during visits to village	65		0.670			
Communication devices to communicate with health facilities	41		0.674			
Allowances and/or incentives received from the Integrated Programme	14		0.673			
Work relationships with health providers in the health centres	97			0.710		
Work relationships with health providers in dispensaries	96			0.825		
Relations as an Integrated Programme MNCH CHW with community leaders	91			0.472		
Level and quality of the training received from the Integrated Programme	90			0.637		
Own transport to provide services	4				0.871	
Own transport to the facility	4				0.882	
Quality of own work	88					0.657
Services people receive in the referred health facilities	79					0.711
Monthly meetings at the facility	78					0.485
% Variance explained		16.93	14.06	12.51	10.35	9.98
Cronbach’s alpha		0.79	0.66	0.60	0.72	0.44
Mean out of 4		3.27	2.18	3.29	0.38	3.00
SD		0.05	0.06	0.04	0.04	0.05

The first factor included the availability of a range of job aides and registers. It had a relatively high score of 3.27 out of 4, with a majority of respondents (over 90 %) satisfied with the availability of these tools. The second factor was a combination of capacity to perform at work and meet needs of the community, as well as support provided including communication devices, supervisory visits and allowances from the Integrated Programme. While a greater majority reported to be satisfied by their capacity to perform and meet community needs (71–81 %), they were dissatisfied by the support provided to them, especially in terms of allowances (14 %), resulting in a moderate mean score of 2.18 out of 4.

The third factor incorporated items on training received from the Integrated Programme and relations at work including those with healthcare providers working at dispensaries and health centres, as well as with community leaders. Almost all respondents were satisfied with work relations (91–97 %), and 90 % reported to be satisfied with the training received. This factor had the highest mean score of 3.29 out of 4, in contrast to the fourth factor related to the availability of transportation for travelling within their communities to provide services and to use when travelling to the health facilities. This fourth factor had a low score of 0.38 out of 4 suggesting a very high level of dissatisfaction among CHWs.

The fifth factor was an amalgamation of items pertaining to service provision and quality of work. CHWs reported being motivated by the services people received when referred to health facilities by them, the quality of their own work and their participation in monthly meetings at facilities. It had a relatively strong mean score of 3 out of 4.

Mean scale scores for each factor were not found to be significantly different between male and female CHWs. Mean scores for the fifth factor (service and quality) were found to be higher among older CHWs than younger CHWs although the difference was not statistically significant (Table 4). The mean score for the 5th factor were also significantly higher for CHWs who had completed primary education as compared to secondary education (Table 5). Findings also showed that satisfaction with availability of job aids was greater among CHWs in the highest asset thirtile (Table 5). No statistical difference was found between socio-demographic characteristics for other factors of job satisfaction.

**Table 4:** Differences between factors of job satisfaction and motivation determinants by sex and age for 228 CHWs working for the Integrated MNCH Programme in Morogoro Region, Tanzania, in 2013

	Sex			Age (in years)			
	Male	Female	*P* value	<25	25–35	>35	*P* value
Job satisfaction							
Job aides	3.3 (0.6)	3.2 (0.8)	0.3417	3.3 (0.7)	3.3 (0.6)	3.3 (0.8)	0.9931
Capacity and support	2.2 (0.8)	2.1 (0.9)	0.2707	2.1 (0.9)	2.3 (0.8)	2.1 (0.9)	0.3739
Job relations	3.3 (0.5)	3.2 (0.6)	0.1792	3.3 (0.6)	3.3 (0.5)	3.3 (0.5)	0.7880
Transport	0.3 (0.6)	0.4 (0.8)	0.2068	0.5 (0.9)	0.4 (0.5)	0.3 (0.5)	0.1061
Service and quality	3.1 (0.7)	2.9 (0.7)	0.1927	2.9 (0.8)	2.9 (0.7)	3.2 (0.6)	0.0211*
Motivation determinants							
Extrinsic	1.9 (0.9)	1.9 (0.9)	0.8678	1.8 (0.9)	1.9 (0.9)	2.0 (0.9)	0.2711
Job availability	2.2 (0.9)	2.0 (0.9)	0.3317	1.9 (0.9)	2.0 (1.0)	2.3 (0.9)	0.0490*
Altruism	3.4 (0.6)	3.2 (0.6)	0.0924	3.2 (0.6)	3.2 (0.6)	3.5 (0.5)	0.0002*
Intrinsic needs	3.2 (0.7)	3.0 (0.7)	0.1109	2.9 (0.8)	3.2 (0.7)	3.2 (0.7)	0.0384*

*Statistically significant, *P* < 0.05

**Table 5:** Differences between factors of job satisfaction and motivation determinants by education and wealth status for 228 CHWs working for the Integrated MNCH Programme in Morogoro Region, Tanzania, in 2013

	Education completed			Asset index (thirtiles)			
	Primary	Secondary	*P* value	First	Second	Third	*P* value
Job satisfaction							
Job aides	3.2 (0.8)	3.3 (0.6)	0.323	3.0 (0.9)	3.4 (0.6)	3.4 (0.5)	0.0026*
Capacity and support	2.1 (0.8)	2.1 (0.9)	0.9727	2.1 (0.8)	2.1 (0.9)	2.3 (0.7)	0.1791
Job relations	3.3 (0.5)	3.3 (0.6)	0.485	3.3 (0.5)	3.3 (0.6)	3.3 (0.5)	0.8288
Transport	0.4 (0.6)	0.4 (0.7)	0.6988	0.3 (0.5)	0.4 (0.7)	0.5 (0.6)	0.1752
Service and quality	3.1 (0.6)	2.9 (0.8)	0.0304*	3.1 (0.7)	2.9 (0.8)	2.9 (0.6)	0.0978
Motivation determinants							
Extrinsic	1.9 (0.9)	1.8 (0.9)	0.1035	1.9 (0.9)	1.8 (1.0)	1.9 (0.9)	0.8000
Job availability	2.2 (0.9)	1.9 (0.9)	0.0317*	2.4 (0.9)	1.8 (1.0)	2.1 (0.9)	0.0015*
Altruism	3.4 (0.5)	3.2 (0.6)	0.0364*	1.9 (0.8)	1.8 (1.0)	1.9 (0.9)	0.1804
Intrinsic needs	3.3 (0.6)	2.9 (0.8)	0.0041*	2.4 (0.9)	1.8 (1.0)	2.2 (0.9)	0.4067

*Statistically significant *P* < 0.05

## Discussion

This study aimed to identify factors associated with motivation and satisfaction among a recently trained and deployed volunteer cadre of CHWs in Morogoro Region, Tanzania. CHWs were more likely to be motivated to become CHWs due to altruism (work on MNCH, desire to serve God, work hard) and intrinsic needs (help community, improve health, pride) than due to external stimuli (monetary incentives, skill utilization, community respect or lack of professional alternatives). Factors strongly associated with CHW satisfaction included relationships with health workers and communities, the availability of job aides and the capacity to provide services and with the training. On the other hand, CHWs were highly dissatisfied with the unavailability of transportation for carrying out their tasks and the lack of allowances or financial incentives. Factors associated with motivation and satisfaction did not substantially differ across socio-demographic CHW characteristics, although CHWs who were older and less educated were more likely to report motivation and job satisfaction.

Findings of this study pertaining to motivation behind becoming a CHW corroborate those previously reported from Morogoro. Qualitative research found that strong altruistic motivation was not incompatible with the importance of financial compensation of some kind, particularly considering the vulnerable livelihoods among these communities [25]. In other settings, for example, in Bangladesh, the reasons respondents chose to become CHWs included a desire to improve community health and to serve women and children and not aspirations for financial independence [26]. Trust-based relationships with rural communities, an altruistic motivation to serve rural people and sound health knowledge and skills were reported to facilitate successful implementation of CHW programmes in Iran [27].

The strongest satisfaction factor for CHWs was related to work relations with varied CHW stakeholders and training. Similarly, a study conducted in Nepal found that one of the most enabling factors for the sustainability and scaling up of CHW programmes was integration of CHWs with the health system and existing healthcare providers [28]. Community support has been found to be critical for making CHWs feel welcome, acceptable and appreciated in the communities where they provide services in various settings [8, 29]. With regard to training, technically strong and relevant training valued by CHWs and respected by communities has been reported to be an important factor for satisfaction, retention and motivation of CHWs in other low- and middle-income countries as well [8, 30–32]. Increased training corresponding to a broader scope of work and, in particular, having more curative tasks may too increase motivation, service delivery time and time spent working [12].

Another strong factor for satisfaction, the availability of job aids and registers, indicated the importance CHWs attach to having necessary supplies to carry out their tasks effectively. Other studies have also reported the availability of job aids as critical, whether in improving the efficiency of their tasks and in supporting communication with beneficiaries [8, 17, 33]. The capacity to provide services and receive support was also a strong satisfaction factor. Studies have documented similar findings reflecting “personal satisfaction about their contribution to the work”, self-efficacy (able to handle tough situations, solve problems, feel emotionally and physically perfect on work) and self-esteem as factors influencing satisfaction [25, 30, 31, 34].

While CHWs reported to be highly satisfied by their capacity to meet needs of the community, they were dissatisfied by the specific types of support that had not been adequately provided to them (allowances, supply of communication devices and transportation). Dissatisfaction with support received, particularly financial compensation, has been found in other low-resource settings [25, 33–37] as well as in the same region in Tanzania in a prior study [38]. Financial incentives have also been reported as dominant motivating factors for retention of these providers [26, 39].

Similar to other settings [39], lack of availability of transport for travelling to households and health facilities also appeared to have caused discontentment among CHWs. This can be attributed to the fact that CHWs in Morogoro Region have to travel across an expansive and difficult terrain, making it both strenuous and time-consuming to complete scheduled household visits without readily available transportation. The programme had promised to give the CHW bicycles to facilitate movement during care provision. However, it was not possible to follow through on this promise in a timely manner, and the survey was conducted before they received them.

### Limitations of the study

The study was conducted as part of a larger study to evaluate the Integrated Programme and the CHWs who worked with it. There is therefore a possibility of desirability bias in the responses received, particularly around altruistic motivations for being a CHW. Attempts were made to reduce this by assuring respondents of confidentiality and by using investigators external to the programme. The study did not interview community members to share their experiences about the performance of CHWs. This could have helped to better understand community perceptions of CHWs’ performance and hence assess strengths and weaknesses of the Integrated Programme. Although scale items for both job satisfaction and motivation were selected based on discussions with key stakeholders and an extensive literature review, additional qualitative approaches to check face and content validity were not carried out. Although these results closely match those obtained from a qualitative study conducted in the same setting previously [38], concurrent qualitative research or further field testing of the items could have resulted in increasing the total variance explained by the scale.

In addition, a global or overall item of motivation or satisfaction was not included in the scale, limiting the ability to measure correlations between different facets of these constructs with an overall score. The grouping of a few items resulting from factor analysis, for example, the fifth factor for job satisfaction, included heterogeneous items, which collectively did not necessarily represent a composite factor. This could provide a possible explanation for the low value of Cronbach’s alpha for this particular factor. Similarly, the fourth factor of motivation (intrinsic needs) determinants also includes unrelated factors. Testing the psychometric properties of the reduced scales obtained for both job satisfaction and motivation in this study could improve further research inquiry in similar settings.

### Implications

This study has significant policy and programmatic implications for the current CHW programme in Morogoro Region, as well as its scale up to other parts of Tanzania and other low- and middle-income countries. The fact that the CHWs were selected from and by their own communities may have contributed to high levels of satisfaction with relations with co-workers and community members. The importance of interpersonal relations with health workers for CHWs is also an important finding, pointing to the importance of elements such as trust in health worker performance [40, 41].

The CHWs interviewed during this study were from a government programme supported by external technical assistance and had access to essential working tools such as job aides and registers to record patient information. The importance CHWs attached to training in this programme, by highly qualified trainers, instilled performance confidence among the trainees. Sustained availability of working tools and training will ensure high satisfaction among the current cadre of CHWs and is likely to attract others into the workforce in the future.

On the other hand, this study suggests that the current programme did not at the time of the survey provide adequate transportation and monetary compensation to CHWs. In order to improve coverage of visits to target households as per the recommended schedule, given the large catchment area under each CHW, the current and future programmes should strive towards ensuring means of transport or equivalent monetary resources to CHWs. While findings suggest that CHWs were more likely to join this profession due to altruism and to meet their intrinsic needs, they became dissatisfied by the lack of financial incentives offered to them. This result could be due to the growing demands on their time, comparisons that may be drawn with other community level cadres that are paid and/or the desire to contribute to their household income – an activity that programme engagement may detract from. Thus, CHW programmes should also advocate for financial incentives for this cadre either in the form of a salary or stipends to influence both job satisfaction and motivation and consequently the attrition rate [38, 42].

### Further research

In 2015, it is anticipated that the government of Tanzania will initiate a national CHW programme. While this study highlights several key determinants of motivation and satisfaction that need to be considered, additional research is warranted to better understand nuances regarding intrinsic vs extrinsic motivations to enlist as a CHW and levels of monetary compensation to ensure their job satisfaction. The relationship between job satisfaction and performance should also be studied in greater detail for this specific context. Future research should also consider further scale refinement and larger sample sizes to support subgroup analyses of interest.

## Conclusion

Study findings suggest that motivation of CHWs in Morogoro Region could be further improved by providing a more holistic combination of financial and non-financial incentives and building on existing altruism and intrinsic needs but also not ignoring financial and other programme inputs. High levels of satisfaction with relationships with health workers and communities, the availability of job aides and the capacity to provide services with training indicate that strengthening these inputs in the programme could be highly beneficial. A high level of dissatisfaction with the lack of transportation options for use during care provision and financial incentives could be potential pitfalls for sustainability of the programme. As efforts gain momentum to rollout a national cadre of CHWs, improved understanding of CHWs as a heterogeneous group with nuanced needs and varied ambitions is vital for ensuring sustainability of the programme.
